# Surgery in Gastric Dilatation

**Published:** 1900-02-10

**Authors:** 


### SURGERY IN GASTRIC DILATATION.

MUCH has been published lately in regard to the diagnosis of the condition of the stomach, its capacity, its muscular contractility, and the digestive activity of its secretions. But, after all, modern methods—stomach bucket, stomach tube, or what not—do not tell us the nature of the disease. All we get is but a more exactly defined set of symptoms; indeed, in many cases of dilatation of the stomach nothing short of actual inspection will tell with certainty what is the matter with that viscus. The question is, at what stage of the malady should such actual inspection be undertaken? In regard to this we would refer to an instructive clinical lecture by Mr. William Bennett,[1] of St. George's Hospital, on dilatation of the stomach considered from the surgical aspect, in which he shows the extreme unreliability of many of the ordinary symptoms as means of ascertaining the condition to which they owe their origin. In regard to the question of treatment one may divide the history of a case of dilatation of the stomach into three stages: (1) That of irritation due to interference with its function, showing itself by pain, occurring at first only when the functions of the organ are most active, that is immediately after taking food. At this stage the symptoms are merely those of irritative dyspepsia. (2) A stage in which there is either (*a*) tolerance, when, although the viscus may contain a large quantity of material, no discomfort is felt beyond abdominal fulness and distaste for food; or (*b*) a condition of more or less constant pain, intensified after food, and often ending in vomiting of partly digested food. (3) Advanced dilatation with recurrent irritability, the result of fermentation, which produces periodical vomiting of large quantities of sour, offensive material. Now, in the first stage, there is clearly nothing to suggest operation, while in the third stage, when the dilatation has been followed by organic changes, such as atrophy in the stomach walls, the chances of a cure even by the most successful operation must be comparatively small. It is in the second of the stages mentioned above that operation seems to be demanded. So long as the symptoms amount to mere discomfort a cure by medical treatment may be sought; but when there is acute pain, mainly in the subhepatic region, which is increased by taking food, Mr. Bennett has no doubt that surgical measures are directly indicated in the form of an exploratory abdominal section, it having, of course, been previously ascertained by careful examination that no hernia or other abnormal condition about the parietes exists which may possibly exercise a harmful effect upon the function of the stomach. In other words, it is most important in this as in other conditions that surgical treatment should not be delayed too long. The extreme cases may fairly be left to be managed by lavage and abdominal massage; but if these means fail, then again surgical intervention is clearly indicated as a means of making life tolerable, although hardly as a hopeful means of cure.

[1] Brit. Med. Jour., Feb. 3.

